# Local Population Structure and Patterns of Western Hemisphere Dispersal for *Coccidioides* spp., the Fungal Cause of Valley Fever

**DOI:** 10.1128/mBio.00550-16

**Published:** 2016-04-26

**Authors:** David M. Engelthaler, Chandler C. Roe, Crystal M. Hepp, Marcus Teixeira, Elizabeth M. Driebe, James M. Schupp, Lalitha Gade, Victor Waddell, Kenneth Komatsu, Eduardo Arathoon, Heidi Logemann, George R. Thompson, Tom Chiller, Bridget Barker, Paul Keim, Anastasia P. Litvintseva

**Affiliations:** a TGen North, Translational Genomics Research Institute, Flagstaff, Arizona, USA; b Informatics and Computing Center, Northern Arizona University, Flagstaff, Arizona, USA; c Mycotic Diseases Branch, National Center for Emerging and Zoonotic Infectious Diseases, Centers for Disease Control and Prevention, Atlanta, Georgia, USA; d Division of Public Health Services, Arizona Department of Health Services, Phoenix, Arizona, USA; e Asociación de Salud Integral, Guatemala City, Guatemala; f Universidad de San Carlos, Ciudad Universitaria, Guatemala City, Guatemala; g Division of Infectious Diseases, Department of Medicine, University of California, Davis, Davis, California, USA; h Microbial Genetics and Genomics Center, Northern Arizona University, Flagstaff, Arizona, USA

## Abstract

Coccidioidomycosis (or valley fever) is a fungal disease with high morbidity and mortality that affects tens of thousands of people each year. This infection is caused by two sibling species, *Coccidioides immitis* and *C. posadasii*, which are endemic to specific arid locales throughout the Western Hemisphere, particularly the desert southwest of the United States. Recent epidemiological and population genetic data suggest that the geographic range of coccidioidomycosis is expanding, as new endemic clusters have been identified in the state of Washington, well outside the established endemic range. The genetic mechanisms and epidemiological consequences of this expansion are unknown and require better understanding of the population structure and evolutionary history of these pathogens. Here we performed multiple phylogenetic inference and population genomics analyses of 68 new and 18 previously published genomes. The results provide evidence of substantial population structure in *C. posadasii* and demonstrate the presence of distinct geographic clades in central and southern Arizona as well as dispersed populations in Texas, Mexico, South America, and Central America. Although a smaller number of *C. immitis* strains were included in the analyses, some evidence of phylogeographic structure was also detected in this species, which has been historically limited to California and Baja, Mexico. Bayesian analyses indicated that *C. posadasii* is the more ancient of the two species and that Arizona contains the most diverse subpopulations. We propose a southern Arizona-northern Mexico origin for *C. posadasii* and describe a pathway for dispersal and distribution out of this region.

## IMPORTANCE

Coccidioidomycosis, or valley fever, is caused by the pathogenic fungi *Coccidioides posadasii* and *C. immitis*. The fungal species and disease are primarily found in the American desert southwest, with spotted distribution throughout the Western Hemisphere. Initial molecular studies suggested a likely anthropogenic movement of *C. posadasii* from North America to South America. Here we comparatively analyze eighty-six genomes of the two *Coccidioides* species and establish local and species-wide population structures to not only clarify the earlier dispersal hypothesis but also provide evidence of likely ancestral populations and patterns of dispersal for the known subpopulations of *C. posadasii*.

## INTRODUCTION

*Coccidioides immitis* and *C. posadasii* are the etiological agents of coccidioidomycosis, or valley fever, a primarily pulmonary disease that causes tremendous morbidity (i.e., thousands of new infections per year) in the southwestern United States and other focal regions in the Americas (1). *C. posadasii* was first characterized as “non-California *C. immitis*” found in the southwestern U.S. states of Arizona (AZ), New Mexico (NM), and Texas (TX) and in Mexico and sporadic locales in Central and South America and was later designated a distinct species, in 2002 (2). *C. immitis* is found primarily in California’s Central Valley and the southern California-Baja, Mexico, region and has been recently described as endemic in southeastern Washington state (3). The two species are similar according to clinical and microbiological phenotyping, although specific differences have been reported (4, 5). *Coccidioides* is a dimorphic fungus, with a saprobic phase consisting of mycelia in the soil and a parasitic phase from inhaled arthroconidia, resulting in mammalian infection (6). Death and burial of the mammalian host can lead to reinfection of the soil (7). Both species of *Coccidioides* follow these stages, and infections by those species appear to lead to similar clinical outcomes (8).

Previous genomic analyses of targeted loci (e.g., microsatellites) led to the understanding that the California populations and the non-California populations are genetically distinct species (2, 9). Whole-genome analysis has supported this concept, with hundreds of thousands of single nucleotide polymorphisms (SNPs) separating the two species (10). In a comparative analysis of a single genome from each species, Sharpton et al. (8) identified 1.1 to 1.5 Mb (approximately 4% to 5%) of dissimilar genetic content in the two genomes. A later comparative genomics study, based on the analysis of 10 genomes of each species, determined that the separation of the two species has not remained fully complete and documented possible presence of introgression from hybridization events following species separation (11). As rapid DNA sequencing analysis has become more accessible to research and public health laboratories, approaches such as whole-genome SNP typing (WGST) have been shown to be useful for identifying clonal fungal outbreaks (12–15). However, genomic epidemiology is also needed to help establish the location of exposure of a nonclonal outbreak and robust phylogenomic analysis is needed for in-depth investigation of the local population structure to better understand pathogen emergence, dispersal, and expansion (15).

It is currently thought that wind, water, mammal hosts, and anthropogenic causes are important mechanisms for local and geographic area-scale dispersal of arthroconidia (16, 17). Using microsatellite data from 163 isolates, Fisher et al. (9) described a phylogeographic *C. posadasii* pattern that was hypothesized to have been driven by human migration, strongly suggesting a movement of *Coccidioides* into South America that was contemporary with the first human movements onto the continent. While that groundbreaking study was critical to understanding large-scale phylogeographic features of *Coccidioides* epidemiology, and while evidence was identified for local population structure, additional microsatellite analysis of Arizona-only isolates was unable to identify a local structure within that state (18).

Given the limited number of *Coccidioides* genomes previously available and the complexities of fungal recombination (19), an accurate understanding of population structure and phylogeography has been problematic. Here we provide comparative phylogenomic and recombination analysis of 86 *Coccidioides* genomes with some insight into the population structure, mutation rates, and phylogeographic patterns between and within the two species, as well as a proposed model for the global dispersal and distribution of *C. posadasii*.

## RESULTS

### Genome sequencing and SNP calling.

**FIG 1:**
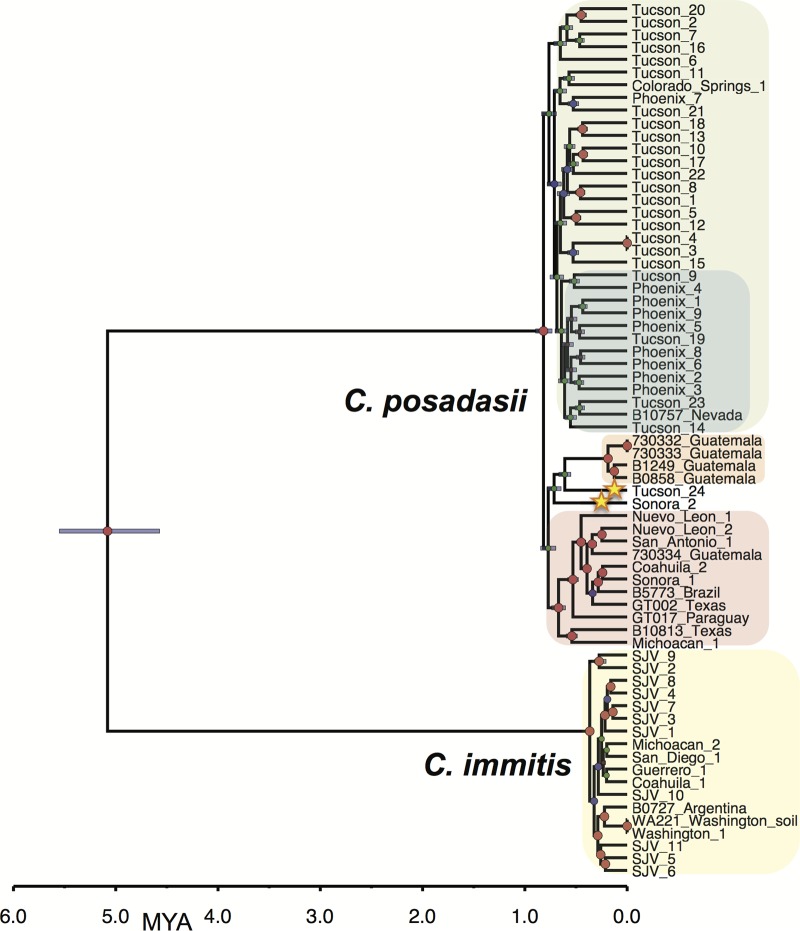
Bayesian phylogenetic analysis of *C. immitis* and *C. posadasii* isolates from all known regions of endemicity. The Bayesian statistical framework incorporated in BEAST 1.8.1 was used to integrate prior information, in the form of internal node timing estimates (from a fossil record) with a rooted tree, to produce a calibrated phylogeny. The analysis was performed on WGS data from 69 *Coccidioides* genomes (18 *C. immitis* and 51 *C. posadasii*). Clades of interest are highlighted as follows: green, Arizona; blue, Phoenix subclade of Arizona; orange, Guatemala; pink, Texas-Mexico-South America; and yellow, *C. immitis*. Stars highlight strains of interest. Posterior probabilities are indicated by node size. Purple node bars are shown for each node and are informative for the 95% confidence interval for the timing estimate. The timeline represents millions of years before the present.

Sixty-eight newly sequenced genomes were compared to 18 of the existing 28 published genomes (11, 12, 14), resulting in a total of 86 genomes (26 *C. immitis* and 60 *C. posadasii*) available for analysis (see Table S1 in the supplemental material). The depth of sequence coverage for the new genomes ranged from 23× to 228× (average, 67×). The two assembled genomes (San Diego_1 and B10813_Tx) used as references had a total of 28,389,157 and 28,389,157 bases, respectively. San Diego_1 assembled into 3,464 contigs with an *N*_50_ of 200,441 bases. B10813_Tx assembled into 2,254 contigs and had an *N*_50_ of 43,956 bases. The publically available Sanger sequencing data http://www.broadinstitute.org/annotation/genome/coccidioides/group/MultiHome.html included only final assemblies, making it difficult to distinguish true SNPs from sequence error; as such, these genomes were not included in all population analyses. Six of the previously published Illumina sequences (11) had low (3.4× to 8.5×) depth of coverage, analyses of the three previously published SOLiD sequences (12) used short reads (35 to 50 bp), resulting in larger error rates (data not shown), and these sets were removed from the analyses. The genus-level SNP analysis included the 69 genomes (18 *C. immitis* and 51 *C. posadasii*) with highest coverage and quality, excluding the previously published genomes to maximize the number of identifiable SNPs. That analysis identified 405,244 shared SNP loci, that is, loci in genetic content shared by all strains (Fig. 1), with 296,632 of those being parsimony informative. A more inclusive phylogenetic analysis, using available Sanger sequenced isolates and including 81 genomes (22 *C. immitis* and 59 *C. posadasii*) (see Fig. S1 in the supplemental material), found only 128,871 shared SNP loci. The total number of SNPs was reduced likely due to filtering out of low-quality SNPs in the previously published Sanger sequence data; however, the overall topologies did not demonstrably change between the conservative and inclusive phylogenies.

Large proportions of the parsimony-informative SNPs (28,660; 40.7%) separate the two species (additional genus-level Bayesian, likelihood, and parsimony trees can be found in the supplemental material). Additionally, an analysis of molecular variance (AMOVA) showed significantly high levels of genomic separation (ΦPT = 0.91, *P* < 0.001), providing clear support for their taxonomical distinction. *C. posadasii* demonstrated a larger average SNP distance (i.e., the number of base substitutions per site from averaging overall pairwise distances between isolates within each species) than *C. immitis* (0.062 versus 0.024, respectively) (Table 1). Furthermore, Bayesian estimations of molecular rates suggest that *C. posadasii* experienced a much earlier within-species divergence than *C. immitis* (Fig. 1 and below).

**TABLE 1:** Summary statistics of *Coccidioides* population analyses

*Coccidioides* population	Avg SNP distance (SEM)	Avg no. of lineage-specific SNPs	Population comparison	Fst	ΦPT
*C. immitis*—all	0.024 (0.004)	12,149	*C. immitis* versus *C. posadasii*^a^	0.905	0.905
*C. posadasii*—all	0.062 (0.009)	4,581	Tucson versus Phoenix	0.043	0.039
*C. posadasii*—Tucson	0.159 (0.080)	13,031	Tucson versus Tex-Mex-SA^a^	0.206	0.210
*C. posadasii*—Phoenix	0.142 (0.063)	12,967	Tucson versus Guatemala^a^	0.287	0.291
*C. posadasii*—Tex-Mex-SA	0.119 (0.051)	9,140			
*C. posadasii*—Guatemala	0.037 (0.014)	4,083			

a Population comparisons with significantly different SNP distances at *P* of <0.001 via analysis of variance (ANOVA).

### *Coccidioides posadasii* population structure.

**FIG 2:**
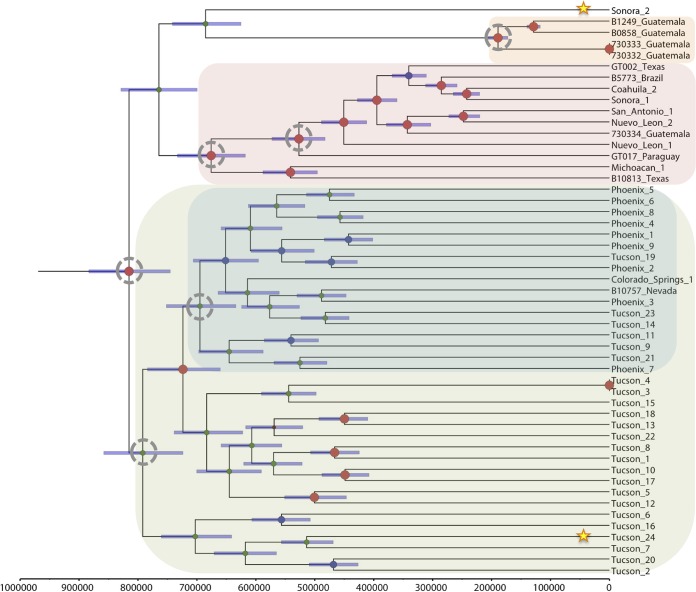
Bayesian phylogenetic analysis of *C. posadasii* isolates. Beast 1.8.1 was used to produce a calibrated phylogeny, where the time to most recent common ancestor estimated for *C. posadasii* from the dual-species analysis whose results are presented in Fig. 1 was used for calibration here (TMRCA for *C. posadasii*, 818,100 years). The analysis was performed on WGS data from 51 *C. posadasii* genomes. Clades of interest are highlighted as follows: green, Arizona; blue, Phoenix subclade of Arizona; orange, Guatemala; and pink, Texas-Mexico-South America. Stars highlight strains of interest, while dotted circles represent TMRCA of interest. Posterior probabilities are indicated by node size. Purple node bars are shown for each node and are informative for the 95% confidence interval for the timing estimate. The timeline represents years before the present.

Phylogenetic histories of each species were interrogated separately, using multiple phylogenetic inference and population genomics tools, as no one tool can provide a complete phylogenomic picture. The *C. posadasii* analysis was primarily based on 51 genomes: 38 isolates from the United States, 6 isolates from Mexico, 5 isolates from Central America (Guatemala), and 3 isolates from South America (Brazil, Paraguay. and Argentina). Isolate locales are based on available metadata and may represent the location of sample isolation, the laboratory that stored the isolate, or the home of the patient (see Table S1 in the supplemental material). A total of 253,291 SNPs from shared loci (134,752 parsimony informative) were identified among the *C. posadasii* isolates. The pairwise homoplasy index (phi) test found statistical evidence for recombination (*P* = 0.0); however, the three major genetic populations were clearly separated and the separation was well supported by standard population statistics (Table 1). Evidence from three Bayesian analyses ([i] BEAST [Fig. 2], [ii] *fineStructure* [see Fig. 4], and [iii] BAPS [see Fig. 5]), as well as from the maximum parsimony and maximum likelihood (ML) bootstrap analyses (see Fig. S3 and S4 in the supplemental material), supports this separation into three geographically related populations: (i) Arizona; (ii) Texas, Mexico, and South America; and (iii) Guatemala.

Within the Arizona population, several subclades were identified by the phylogenetic analyses and the identifications were well supported by the ML bootstrap analysis (see Fig. S4 in the supplemental material). One subclade included all isolates from the central Arizona (i.e., Phoenix) region as well as a small number of strains from Tucson, AZ, and the other two subclades were primarily comprised of the isolates from the Tucson region. However, the low Fst (0.043) and ΦPT (0.039) values and analyses using population genetics methods (see Fig. 4 and 5) suggest that these geographically distinct subpopulations are not genetically distinct.

The second *C. posadasii* population included almost all isolates from Texas, Mexico, and South America, except for the Sonora_2 strain, from northern Mexico, which appeared to be basal to this group in the maximum parsimony and neighbor network analysis (see Fig. S3 and S5 in the supplemental material). The third population of *C. posadasii* included four Central American isolates from Guatemala that formed their own distinct population, supported by all analyses. A fifth Guatemalan isolate (i.e., isolate 730334) was collected from a patient who was infected while traveling in Texas, which is consistent with the placement of this isolate in the Texas-Mexico-South America (Tex-Mex-SA) population in all phylogenetic analyses.

In addition, two strains, Sonora_2 and Tucson_24, appeared as distinct early lineages in the non-Arizona clades. Furthermore, when both *C. posadasii* and *C. immitis* isolates were included in the analyses, Bayesian as well as maximum likelihood methods identified the Sonora_2 and Tucson_24 strains as possible basal lineages for the Guatemalan population (Fig. 1; see also Fig. S4 in the supplemental material). However, when only *C. posadasii* strains were included in the Bayesian analysis, the Tucson_24 strain was placed within the Arizona population, while the Sonora_2 strain remained basal to the Guatemalan clade (Fig. 2). Multiple phylogenetic methods using the same nucleotide substitution model produced phylogenies with slightly differing results, suggesting that the observed incongruences are an artifact of differing algorithms implemented in differing phylogenetic methods.

### ***Coccidioides immitis***.

**FIG 3:**
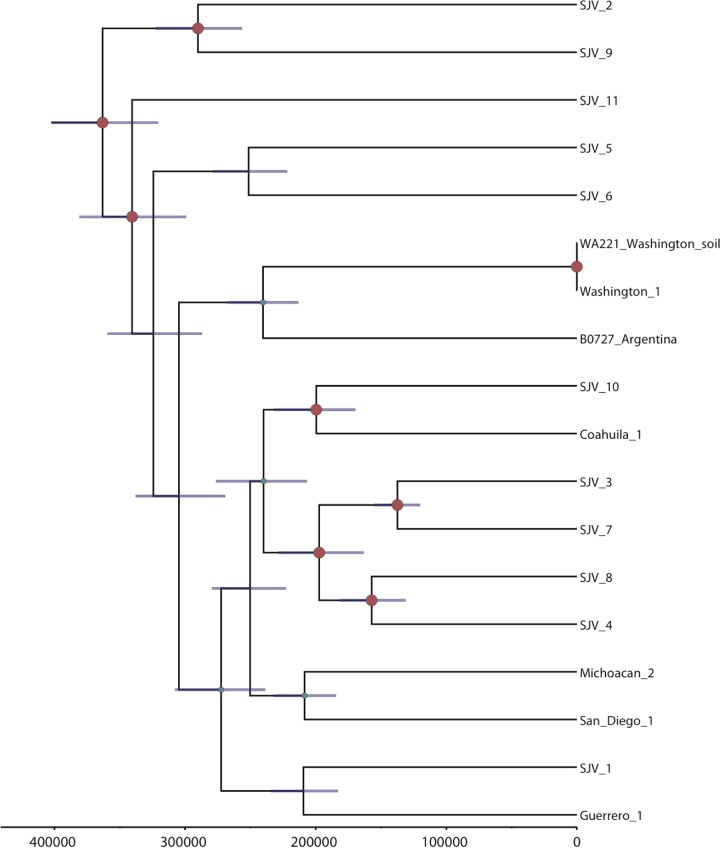
Bayesian phylogenetic analysis of *C. immitis* isolates. Beast 1.8.1 was used to produce a calibrated phylogeny, where the time to most recent common ancestor estimated for *C. immitis* from the dual-species analysis whose results are presented in Fig. 1 was used for calibration here (TMRCA for *C. posadasii*, 365,700 years). The analysis was performed on WGS data from 18 *C. posadasii* genomes. Posterior probabilities are indicated by node size. Purple node bars are shown for each node and are informative for the 95% confidence interval for the timing estimate. The timeline represents years before the present.

We identified 64,096 shared SNP loci (31,372 parsimony informative) among 18 analyzed *C. immitis* genomes. Bayesian analysis (Fig. 3), BAPS analysis (see Fig. S6 in the supplemental material), maximum likelihood analyses (see Fig. S7), and maximum parsimony analysis (see Fig. S8) each displayed similar overall topologies for *C. immitis*, where each subpopulation or clade generally contained at least one member from the Central Valley of California (i.e., San Joaquin Valley). The Washington state (WA) and Argentina isolates typically grouped together. The Argentinian isolate came from a patient that was treated in Buenos Aries (not thought to be an site of endemicity and greater than 1,200 km from the nearest known zone of endemicity for *C. posadasii*) and possibly represents an exposure outside Argentina (although autochthonous South American exposures to *C. immitis* have been recently reported [20]). Washington was previously shown to have recently become a region of endemicity for such isolates (3) (and the isolates were shown to be a distinct genetic subpopulation by BAPS analysis—see Fig. S6). The strength of all of these analyses, however, is limited due to smaller number of *C. immitis* genomes used in this study.

### Population genomics of and mating type distribution of *Coccidioides.*

**FIG 4:**
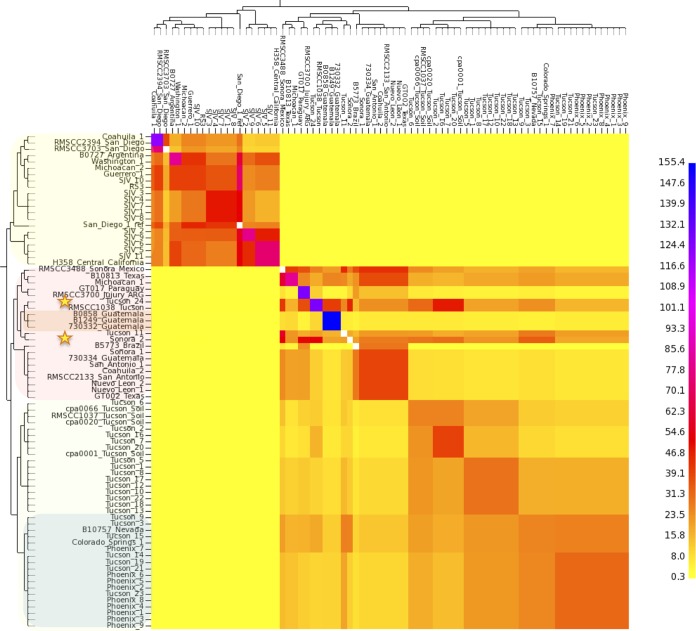
Genome-sharing analysis of *Coccidioides* species complex. *fineStructure* analysis was performed using the SNP matrix developed for Fig. S1 in the supplemental material. The SNPs from 66 *Coccidioides* genomes were reduced to a pairwise similarity matrix, which was used to identify population structure based on shared haplotype regions of genome. The *x* axis analysis represents the strain as a “recipient” of genomic regions, and the *y* axis represents the strain as a “donor” of genomic regions. The scale bar represents the number of shared genome regions, with blue representing the largest amount of sharing and yellow representing the smallest. The shading of isolates on the *y* axis correlates with clades in Fig. 1.

**FIG 5:**
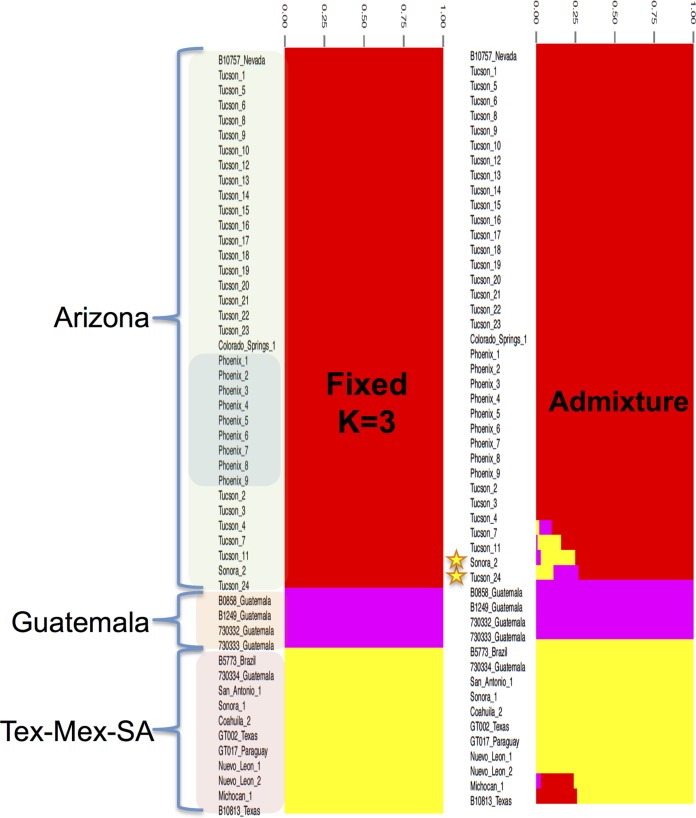
Population structure analysis of *C. posadasii*. Bayesian analysis of *C. posadasii* population structure was carried using BAPS 6.0, 3 fixed genetically diverged groups previously established by phylogenetic inferences (see Fig. S2 in the supplemental material), and 10 replicates. Admixture graphs of three identified *C. posadasii* mixtures (populations) were plotted using 200 simulations, and the percentage of genetic composition from each isolate was plotted. The shading of isolates in left column correlates with clades shown in Fig. 1.

In order to explore previously identified genetic recombination (21, 22) in the population analysis, we applied *fineStructure* and BAPS, which are model-based Bayesian approaches (23, 24) for incorporating recombination signal into population structure. The genus-level *fineStructure* analysis appropriately separated 81 *Coccidioides* genomes into the two species and assigned within-species “populations,” or groups related by shared genomic regions, which were similar although not identical to those described by other methods (Fig. 4). Genomic isolation (shown as yellow on the heat map) was demonstrated for the two species; however, shared genetic history (shown by a gradual coloration on the heat map) was seen among members within each species. Several *C. posadasii* isolates displayed genomic space shared with members from outside their respective assigned groups, indicating the presence of historical admixture or incomplete (or recent) separation between groups within the species (Fig. 4 and 5). Notably, evidence was provided for an extensive genome-sharing history of both the Tucson_24 and Sonora_2 strains with the remainder of the *C. posadasii* isolates, providing additional evidence that these genomes are representative of older, more ancestral lineages and explaining why different analyses group these strains differently (note that the 1038_Tucson strain appears to be closely related to Tucson_24; however, this is one of the previously Sanger-sequenced strains, and it is therefore not included in all analyses). The BAPS admixture analysis determined that admixture was present in these two strains, along with Tucson_11 and Tucson_7, as well as in B10813_Tx and Michoacán_1 (Fig. 5). Given the geographic separation, the identified admixture is likely indicative of lineage history rather than recent recombination between subpopulations. Both *fineStructure* and BAPS analyses highlighted the relative isolation of the Guatemalan clade, as well as limited interpopulation genome sharing of the individual strains from Brazil, Paraguay, and Argentina, with the latter two countries displaying the most between-strain genomic sharing outside the Guatemalan group.

The population structure of *C. immitis* reveals strong genetic isolation of WA isolates. In addition, there is low genetic variation within the WA clade as represented by short internal branches. This characteristic is unique to the WA clade and is not shared within any other *C. immitis* or *C. posadasii* populations, where each isolate appears to represent a single haplotype. Because recombination has been frequently reported for *Coccidioides*, we assessed the mating type distribution in the main clades of *Coccidioides*. The majority *C. immitis* and *C. posadasii* clades displayed similar ratios of the *MAT1-1* and the *MAT1-2* idiomorphs, suggesting that random mating is possible (chi-square test, *P* values of >0.05) (Fig. S9 in the supplemental material). However, we detected only a single idiomorph (*MAT1-2*) among six WA genomes sequenced so far, which is compatible with a clonal population. The uneven *MAT* idiomorph distribution was evidenced by a significant chi-square test (*P* = 0.0143).

### Divergence and most recent common ancestor.

Divergence analysis was calibrated using two previous estimates for the separation of the two species: 5.1 million years ago (MYA) (8) and 12.8 MYA (9). This analysis suggests that Arizona and non-Arizona *C. posadasii* subpopulations diverged between 820 thousand years ago (KYA) and 2.06 MYA and that the *C. immitis* populations diverged between 370 and 920 KYA, using the 5.1 MYA and 12.8 MYA calibration points, respectively. As the 5.1 MYA speciation estimate was derived from fossil data (8) rather than being inferred from microsatellite mutation rate data, as was the case for the 12.8 MYA estimate (9), it was selected as the calibration point for estimating all other within-species divergence times (Table 2 and Fig. 2). The time to most recent common ancestor (TMRCA) of the Tex-Mex-SA population was estimated at 675 KYA (95% confidence interval [CI], 617 to 733 KYA), the first South American emergence is calculated at 527 KYA (95% CI, 483 to 573 KYA), and the Guatemalan MRCA emerged less than 190 KYA (95% CI, 172 to 206 KYA). Using the combined runs for each species, respectively, and the 5.1 MYA calibration point, the *C. posadasii* estimated mutation rate was calculated to be 1.08 × 10^−9^ SNPs per base per year (95% CI, 9.9237 × 10^−10^ to 1.1752 × 10^−9^), and the estimated *C. immitis* mutation rate was 1.02 × 10^−9^ (95% CI, 9.0797 × 10^−10^ to 1.1431 × 10^−9^), similarly to previous estimated mutation rates for fungi (11, 25).

**TABLE 2:** Estimated time to most recent common ancestor for *Coccidioides* subpopulations, based on 5.1 MYA speciation calibration point

*Coccidioides* population	Estimated TMRCA (yrs ago)	TMRCA 95% confidence interval
*C. posadasii*—all	818,000	740,000–900,900
*C. immitis*—all	365,600	327,500–404,900
*C. posadasii*—Arizona	791,900	723,700–858,000
*C. posadasii*—Phoenix	694,900	633,800–755,900
*C. posadasii*—Tex-Mex-SA	675,900	617,800–733,100
*C. posadasii*—Guatemala	189,200	172,500–205,600

## DISCUSSION

Here we present results of a whole-genome SNP analysis of *Coccidioides* strains from geographically diverse locations that has further confirmed the presence of two species, *C. immitis* and *C. posadasii*, first established by Fisher et al. (2) and further described in subsequent studies (4, 11). Using multiple methods of population and phylogenetic analyses, we provide evidence of further genetic subdivision within the major clades of *C. posadasii* and *C. immitis* and demonstrate the presence of genetically distinct subpopulations associated with specific geographic locales. Calibrated Bayesian analysis provides a new understanding of the sequence of divergence events, particularly within *C. posadasii*. These data and analyses enhance our ability to conduct genomic epidemiology, make more-informed hypotheses of likely ancestry and pathways of dispersal, and better understand the changing distribution of *Coccidioides*.

The genetically differentiated Arizona and non-Arizona *C. posadasii* populations, first proposed by Fisher (9), are statistically distinct, and a third, previously unknown genetically isolated population in Guatemala has now been identified. We are now able to identify multiple *C. posadasii* clades within Arizona and differentiate between isolates from the two major urban areas of Tucson (southern Arizona) and Phoenix (central Arizona), with the Phoenix isolates grouping in a single clade, although without forming a genetically isolated population (Fig. 2, 4, and 5). Such fine-scale resolution of subpopulations allows a better understanding of the originating source or location of cases in areas of nonendemicity and recent emergences in new geographical regions. For example, epidemiological evidence strongly suggests that the case involving the Guatemala_730334 strain was not the result of a Guatemalan exposure but was rather the result of an exposure in Texas, a supposition borne out by the genomic data. In the same respect, the Colorado Springs_1 case was not likely the result of a Colorado exposure, as there is no known area of coccidioidomycosis endemicity in that region of Colorado. However, whole-genome SNP analyses place this strain within the Tucson/Phoenix subclade of the Arizona population of *C. posadasii*, likely representing the originating environmental source for that infection; unfortunately, no travel history from this case was available for our study. In the same respect, the *C. immitis* cases from lower Mexico (Guerrero and Michoacán) and Argentina (Buenos Aires), locales that are not known to be areas of *C. immitis* endemicity (26, 27), are possibly epidemiologically linked to their phylogeographic placement within one or more central California (e.g., San Joaquin Valley) clades. Such phylogeographic associations still require confirmation with local environmental sampling. For example, while the Washington isolates appear to fall within a larger San Joaquin Valley clade, it is now understood, as the result of careful examination of both clinical and environmental isolates, that this clonal population emanates from a new focus of endemicity in southeastern Washington state (3, 14), with possible original dispersal out of California’s Central Valley.

The resolution provided by whole-genome SNP analysis also provides for an understanding of the possible ancestry of this fungus. Radiation of *C. posadasii* is estimated to have occurred ~450K years before the emergence of *C. immitis* (Fig. 1 and Table 2). This is clear evidence of an older and more-diverse population, with *C. posadasii* likely being ancestral to *C. immitis*. Neafsey et al. (11) established that *C. posadasii* has a 2-fold-larger effective population size than *C. immitis*, suggesting that this was due to the larger geographic range, allowing the development of more subpopulations. This, however, may also be explained by *C. posadasii* being an older population, with more time for mutation and divergence. It is of interest to consider that the center of diversity for *C. immitis* is found in the Central Valley of California, a geographic location born of a large inland sea that began retreat during the Pleistocene (28) and most recently was completely submerged by glacial runoff as long as 700 thousand years ago (KYA) (29). The estimated MRCA for *C. immitis* is 365K to 920K years ago, depending on the calibration model, which was subsequent to, or possibly coincided with, the draining of the California (CA) Central Valley. Isolation within glacial refugia has been proposed as a paleogeological factor impacting the historical continental dispersal of environmental fungi (30). The Sierra Nevada mountain range on the east side of the Central Valley, known to have been part of multiple glaciation events, could therefore have played a role as a geographic population barrier and a source for glacial refugia that subsequently drained into the Central Valley, providing a possible paleogeographic cause for a speciation event.

Previous studies provided evidence of introgression between *C. posadasii* and *C. immitis*, which may have originated from a possible hybridization event in some southern California and Baja, Mexico, isolates of *C. immitis* (11). In this study, we observed limited cross-species homoplasy, no identifiable genomic sharing (Fig. 4), and an Fst value of >0.9, all of which are consistent with a limited population effect from hybridization. These data indicate that the two populations are largely reproductively isolated outside the southern California region where suspected hybridization events are thought to have occurred (11). However, as the genomes from those previously identified introgressed strains were not of quality comparable to that of the genomes studied here, they were not analyzed in this study. Further evaluation of hybridization events and patterns of introgression is warranted.

The Arizona clades of *C. posadasii* have an estimated MRCA approximately 115K years before the Tex-Mex-SA population MRCA (Fig. 2), strongly suggesting that the Arizona group diverged earlier than the non-Arizonan *C. posadasii* populations, although there is a small overlap in confidence intervals (Table 2). A possible mechanism for the increased diversity within the Arizona-specific groups is increased recombination; however, only limited evidence of within-haplotype sharing was seen with the *fineStucture* analysis (Fig. 4), and each strain in this population corresponded to a single haplotype according to BAPS analysis (Fig. 5). When analyzed by itself, no admixture was identified within the Arizona population (data not shown), further suggesting that limited recombination is present among the genomes assessed. This limited genetic sharing may be a result of restricted recombination or historical mating events.

The two mating type loci were seen in roughly similar proportions in all geographic areas, except Washington (see Fig. S9 in the supplemental material), suggesting that sexual recombination is possible throughout most of the endemic range. However, *Coccidioides*, like other fungal genera, may be impacted by a number of recombination/mating restrictions, both extrinsic (e.g., geographic separation) and intrinsic (e.g., lack of a need for regular sexual reproduction due to an effective asexual mitosis strategy) (19). No laboratory-controlled genetic recombination has been accomplished with *Coccidioides* to date (31), and the role of, and barriers to, sexual reproduction in the natural setting remains unknown, similarly to other environmental pathogenic fungi (30). The high genetic diversity observed within populations, combined with observed homoplasy but limited recombination detected, is more likely explained as incomplete lineage sorting. Ancestral polymorphisms likely remained through divergent events, accounting for various alleles among related isolates but concurrent alleles in less closely related isolates (32). This would account for the extensive branch lengths, high homoplasy, and little recombination seen among populations and individuals in the parsimony analyses.

Multiple clades occurring in southern Arizona (i.e., Tucson), including one derived “younger” clade that contains all the central Arizona (i.e., Phoenix) isolates, indicates that the southern Arizona area is likely the source of the central Arizona population (Fig. 2). In addition, maximum likelihood analysis indicates that the Tex-Mex-SA and Guatemalan populations have also diverged from the southern Arizona population (see Fig. S2 in the supplemental material); however, results obtained using other methods of phylogenetic reconstruction do not immediately support these relationships (see Fig. S1). A closer look at select strains may provide additional clues to the ancestral source of these populations. The Tucson_24 isolate (along with the Sanger-sequenced 1038_Tucson isolate) and the Sonora_2 (northern Mexico) isolate were placed within the non-Arizona clades in multiple Bayesian analyses (Fig. 1, 4, and 5) and may be ancestral to at least the Guatemalan population. The Sonora_2 isolate and the B10813_Tx isolate appear to be the most basal members of the Tex-Mex-SA clade in the “neighbor-net” tree (see Fig. S5), although they have greatly diverged from each other. The same Texas isolate (B10813_Tx) and the Michoacán_1 (Mexican) isolate are the two most basal to the Tex-Mex-SA clade in the Bayesian tree analyses (Fig. 1 and 2) and in the maximum parsimony tree (where the Guatemala clade was used at the root) (see Fig. S3). Additionally, the Tucson_24 and Sonora_2 isolates displayed the highest levels of admixture with all populations (Fig. 5) and appeared to have the most “shared” haplotype space among all *C. posadasii* isolates in the *fineStructure* analysis of this clade (Fig. 4), suggesting, perhaps, an ancestral genomic background for the species. The South American isolates appear to be derived in the Tex-Mex-SA clade. The Argentina (RMSCC_3700) and Paraguay (GT_1078) isolates are closely related to each other and are closer to other Mexico strains than the Brazil strain (B5773), as shown by both SNP distance and haplotype sharing analyses, suggesting two or more independent founder events in South America.

The Guatemala clade is a genetically distinct subpopulation and emerged much later than the Tex-Mex-SA population. While tropical Guatemala would typically be considered to be outside the arid regions of endemicity, multiple accounts of endemic transmission have been recorded in its arid Motagua River valley (27, 33). These data lend support to the idea of the existence of a local distinct population of *Coccidioides* in Central America, with more recent divergence between individuals than has been seen in other locales. Evidence exists for an additional Central America population in the arid Comayagua Valley in Honduras (1, 34), although no isolates were available for analysis in this study.

The phylogenetic clades or populations identified in the differient geographic regions likely reflect single or limited founder population events, followed by local evolution. Such events would conflict with the hypothesis of ongoing deposition of spores by wind as a likely mechanism for large-scale geographic dispersal (17), as continual dispersal would result in multiple distinct populations in each locale. For example, the grouping of central Arizona (Phoenix) isolates largely clustered in a single subclade of the southern Arizona population. The limited presence of Phoenix isolates outside this clade may represent instances of wind dispersal and/or exposure (i.e., infection in Tucson patients by wind-borne Phoenix-originating *Coccidioides* spores), although it is more plausible that patients infected in Phoenix are occasionally diagnosed in Tucson, and vice versa, as there is a high degree of travel between the two population centers. Cases of patients living in one area of endemicity but having exposure in another area of endemicity have been well documented (21, 35).

A more likely hypothesis is that primary dispersal over large regions occurs through movement by mammalian hosts, similarly to Fisher’s previously proposed mechanism for emergence of *C. posadasii* in South America during the Great American Biotic Interchange (GABI) via the Central America land bridge between the continents (9). The *Coccidioides* genomic adaptations to mammalian hosts (e.g., expansion of protease and keratinase gene families) (8, 36) and the hypothesis that the patchy distribution of *Coccidioides* in soil is due to the fungal association with mammal carcasses (i.e., dead hosts) and burrows (31, 37) comport with a theory that distribution is related to distinct movement events of infected animals. Although multiple North American mammal species can be infected, rodents, canids, and humans are highly susceptible to succumbing to the disease, allowing infecting strains to reenter into the soil upon death (1, 38), and therefore may be considered the most likely “vectors” of transmission of *Coccidioides* from one locale to another. In South America, llamas and armadillos (39, 40) are additionally considered highly susceptible, with the latter being a known source for soil contamination and human exposure (41). The ecological importance of recent findings of infected bat populations in Brazil remains unknown (42).

The TMRCA analysis would suggest a separation of the *C. posadasii* subpopulations over the previous 800K years, dispersal into South America no earlier than 575K years, and emergence in Guatemala less than 200K years ago. A closer look at the timing and composition of the known GABI events compared to the timing of the *C. posadasii* southern dispersions is warranted. There are recorded limited mammal interchanges between the North and South American continents that occurred as early as 9 MYA (43), however, animal exchanges did not begin in earnest until after the final settling of the Central American isthmus about 2.8 MYA (44). Starting about 2.6 MYA, there were four significant exchanges of mammals (i.e., GABI 1 to 4) between North America and South America, typically separated by glacial retreat and other geoclimatic events (43). Humans are understood to have appeared in the Western Hemisphere and migrated to South America well after the last of the GABI events, likely less than 15,000 years ago (45). The earliest estimated emergence of *C. posadasii* in South America (TMRCA = 527 KYA) would have followed the GABI 3 event of multiple carnivores (including canid taxa) emigrating from North America to South America approximately 0.8 M to 1.0 MYA. The final GABI event (~125 KYA) occurred subsequent to the establishment of the Guatemala *C. posadasii* population (TMRCA = ~189 KYA). Of note, the earliest identification of the genus *Canis* in South America occurred at this time, during GABI 4. The establishment of *C. posadasii* in Central America just prior to the GABI 4 event may then be related to the Central American “holding-pen” concept, where migrant mammalian taxa are thought to have established local populations and undergone provincial evolution prior to continuing their southward or northward expansions (43). Such an occurrence could account for a regional establishment of a distinct population of *C. posadasii* at this time. It is also possible that *Coccidioides* was more prevalent throughout Central America during the late Pleistocene epoch, where records show a vastly different climate and biotic landscape of very dry thorn scrub forests and grasslands (46). The current known distribution of *C. posadasii* in Central America is in the dry Motagua River valley of Guatemala and the dry regions of the Comayagua Valley of Honduras (33), perhaps the remaining vestiges of the historically dry *Coccidioides*-supporting landscape in the region. Lastly, the data presented here strongly suggest that humans were not the primary driver of *Coccidioides* dispersal into Central and South America, as had been previously postulated (9).

**FIG 6:**
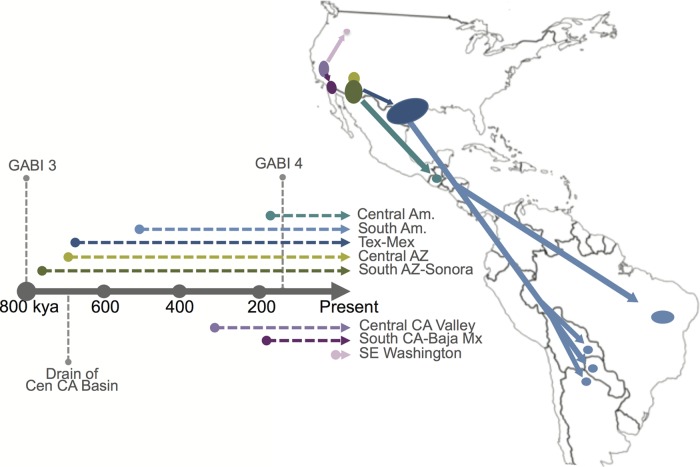
Model for *Coccidioides* dispersal in the Western Hemisphere. A proposed dispersal model for *C. posadasii* from a hypothetical founder population in southern Arizona (South AZ)-northern Mexico and *C. immitis* from a hypothetical founder population in California’s Central Valley (Central CA Valley) is shown in conjunction with a timeline (labeled in thousands of years ago [kya]) that is color coded to match dispersal paths and annotated with the timing of great American biotic interchange (GABI) events 3 and 4, as well as of the final draining of the Central California basin. Central Am., Central America; South Am., South America; SE, southeastern; Mx, Mexico.

An updated *C. posadasii* dispersal model (Fig. 6) would suggest that (i) the central Arizona clade originated from one of southern Arizona subpopulations, with all Arizona populations being 700,000 to 800,000 years old; (ii) Texas and Mexico populations also came from a founding population from the southern Arizona-Sonora region, likely 675,000 to 700,000 years ago; (ii) Mexico (and possibly Texas) subsequently fed the South American populations (likely more than once), as early as 525,000 years ago; and (iv) the Guatemalan population independently, and more recently (<200,000 year ago), emerged from the southern Arizona-Sonora region. The evidence of historical admixture of both the Tucson_24 and Sonora_2 strains with the rest of the species, and the possible basal nature of the Sonora_2 and/or Tucson_24 strains with respect to the Tex-Mex-SA and Guatemalan populations, suggests that these strains are from older lineages and could represent a historical southern Arizona-northern Mexico origin for the species.

While no dispersal model for *C. immitis* is specifically proposed at this time, it is clear that much of the known diversity is found in the Central Valley of California (i.e., the San Joaquin Valley), with the Washington clone being the likely most recent emigrant from this region. The Central Valley being historically submerged under inland sea and fresh water glacial lakes, and the final draining of this valley occurring an estimated 700K years ago, precedes the likely *C. immitis* species MRCA. Additional isolates from outside this region will need to be analyzed to better understand the genomic and dispersal history of this species.

We recognize that the limitations of this study included sampling bias, especially with regard to analyzing fewer *C. immitis* and South American *C. posadasii* genomes, and hence a possibility for both over- and underrepresentation of isolates from some geographic regions. Further conclusions regarding the *C. immitis* population structure should be drawn only on the basis of the inclusion of additional genomes from more locales. The vast majority of the isolates studied here originated from clinical samples. It is possible that environmental soil isolates would provide genotypes differing from those of infecting strains, although this was not seen in previous analyses (7) or specifically seen with the inclusion of previously sequenced genomes from limited *C. posadasii* soil isolates (see Fig. S1 in the supplemental material). Additionally, carefully collected environmental isolates would provide a more reliable representation of locally present strains (31). It is clear that future studies will require a more in-depth analysis of environmental and clinical isolates. It is also important that early sequence data sets likely have less accuracy and coverage, affecting their utility for whole-genome analyses, such as the ones conducted here. Current sequencing methods provide excellent depth and quality that should allow their use for studies well into the future. Numerous phylogenetic tools were employed to improve our understanding of the relationships between individuals and groups. It is understood that no single approach likely represents absolute truth, particularly for a dimorphic fungus with cryptic reproduction; however, the combination of phylogenetic representations provided advances our understanding of potential historical relationships. Likewise, results from the use of Bayesian tools to determine mutation rates and times to the most recent common ancestor do not likely represent actual truth; however, rather than using an earlier calibration based on estimated microsatellite mutation rates, we employed a fossil-derived estimated calibration point for original speciation to provide a most likely representation of specific divergence points. Additionally, we chose to infer molecular rates and divergence times from this calibration point rather than inferring mutation rates and coalescence from tip-dated sequences, the use of which has been suggested to introduce positive bias (47). As more genomes come to be sequenced from the regions of interest in this study and as additional calibration points are established (e.g., from ancient DNA analysis), findings with respect to the time to the most recent common ancestor should continue to be reassessed.

The findings of this study provide a strong argument for additional large-scale population-level sequencing of *Coccidioides*, particularly for both clinical and soil isolates from underrepresented areas, especially Sonora/northern Mexico populations, to further understand the geographic extent of a possible southern Arizona-northern Mexico founding population. Even more importantly, our data demonstrate that a local population structure does occur, even in recombining organisms, and that WGS approaches can be readily used not only for fungal molecular epidemiology of suspect clonal outbreaks but also for linking cases to likely exposure sites and better understanding the patterns of emergence and dispersal.

## MATERIALS AND METHODS

### Isolates selected for sequencing.

Isolates of 50 *C. posadasii* and 18 *C. immitis* were selected from multiple repositories among the collaborators in this study to include primarily geographic diversity (see Table S1 in the supplemental material) and extensive samples from regions of high disease burden (i.e., southern Arizona). Isolate locales are based on available metadata and may represent the location of the sample isolation, the laboratory that stored the isolate, or the home of the patient.

### Genome sequencing.

The genomes of 18 *C. immitis* and 50 *C. posadasii* isolates were sequenced using Illumina HiSeq and MiSeq sequencing platforms, as previously described (14, 48). High-molecular-weight DNA was extracted using a ZR Fungal/Bacterial DNA Mini prep kit (catalog number d6005; Zymo Research, Irvine, CA). DNA samples were prepared for paired-end sequencing using a Kapa Biosystems library preparation kit (catalog number kk8201; Kapa Biosystems, Woburn, MA) protocol with an 8-bp index modification. Briefly, 2 µg of double-stranded DNA (dsDNA) sheared to an average size of 600 bp was used for the Kapa Illumina paired-end library preparation, as described by the manufacturer. Modified oligonucleotides (Integrated DNA Technologies, Coralville, IA) with 8-bp indexing capability (49) were substituted at the appropriate step. Prior to sequencing, the libraries were quantified using quantitative PCR (qPCR), a 7900HT system (Life Technologies Corporation, Carlsbad, CA), and a Kapa library quantification kit (catalog number kk4835; Kapa, Woburn, MA). Libraries were sequenced to a read length of 100 bp or 150 bp on an Illumina HiSeq system or to 250 bp on an Illumina MiSeq system. All WGS data files have been deposited in the NCBI Sequence Read Archive.

### Genome assembly.

The San Diego_1 (*C. immitis*) and B10813_Tx (*C. posadsasii*) sequenced genomes were both *de novo* assembled using the SPAdes assembler (v2.5.0) (50). The San Diego_1 assembly was used as the reference for the “All Species” and *C. immitis* SNP matrices, and the B10813_Tx assembly was used as the *C. posadasii* SNP matrix (see “SNP variant detection” below).

### SNP variant detection.

Illumina read data were aligned against the respective reference assemblies using Novoalign 3.00.03, and then SNP variants were identified using GATK Unified Genotyper v2.4 (51). SNP calls were then filtered using a publically available SNP analysis pipeline (http://tgennorth.github.io/NASP/), as previously described (48), to remove positions that had less than 10× coverage or less than 90% variant allele calls or that were identified by Nucmer as being within duplicated regions in the reference. SNP matrices were produced for the “all species,” *C. posadasii*, and *C. immitis* analyses.

### Phylogenetic analysis.

To understand relationships between isolates, we conducted multiple phylogenetic analyses, including parsimony, likelihood, and Bayesian inference analyses, using whole-genome SNP matrices from a total of 86 genomes (60 *C. posadasii* and 26 *C. immitis*), which included newly sequenced and previously published genomes (see Table S1 in the supplemental material). Maximum parsimony SNP trees, based on each of the SNP matrices, were constructed, using PAUP* v.4.0b10 (52), and were visualized in Fig tree v.1.3.1 (http://tree.bio.ed.ac.uk/software/figtree/). The maximum parsimony all-species *Coccidioides* tree was midpoint rooted and used the San Diego_1 isolate as a reference. The maximum parsimony *C. immitis* tree was rooted using the Coahuila_1 isolate, based on its basal position in the all-species tree, and the San Diego_1 isolate was used as the alignment reference. The maximum parsimony *C. posadasii* tree was rooted using the Guatemala clade, again based on its basal position in the all-species tree, and the B10813_Tx isolate was used as the alignment reference. Due to likely recombination present in the SNP data, a neighbor joining split-tree network or “neighbor-net” tree (53) was drawn to visualize genome sharing between isolates in each of the species trees. The neighbor-net tree was drawn using SplitsTree4 (54) with the uncorrected *P* distance transformation, as previously described (48). Maximum likelihood trees were produced with a rapid and effective stochastic algorithm implemented in IQ-Tree (55). The inferred nucleotide substitution models were TIM3 plus ASC plus R10 for *C. posadasii* and TVM plus ASC plus R7 for *C. immitis*, and 1,000 nonparametric bootstrap pseudoreplicates were performed for branch confidence (56). BEAST v1.8.0 (57) and BAPS (24) were used to infer population structure and produce Bayesian phylogenetic trees, and BEAST was additionally used for mutation rate analysis and time to most recent common ancestor (TMRCA) analysis (see a description of BEAST analyses below). Due to incongruences observed in comparisons of the Bayesian, maximum likelihood, and maximum parsimony trees, we separately compared the models that were used in the BEAST Bayesian analysis and the IQ-Tree maximum likelihood analysis in order to test the effect of model selection on the phylogenetic topologies. The maximum likelihood trees produced using the HKY plus G model and the GTR plus G model were identical and demonstrated the same phylogenetic incongruences compared to the BEAST trees (data not shown). The observed phylogenetic differences were therefore more likely a product of differing methods rather than being attributable to the nucleotide substitution model selection.

For the BAPS analysis, population assignments were assessed using the “fixed-K model.” The numbers of populations (genetic mixture) were tested from K = 2 to K = 15 for *C. posadasii* and K = 2 to K = 5 for *C. immitis* using 10 replicates for each tested K. Analyses of *Coccidioides* admixed ancestry from previously inferred partitions (K = 3 for both species) were conducted using 200 iterations. Admixture results were bar plotted with help of STRUCTURE PLOT (58). Additionally, we tested the Arizona population of *C. posadasii* separately to assess admixture occurring within this population by the use of BAPS analysis. No evidence of admixture or recombination within this population was identified (data not shown). *fineStructure* population analysis (23) was implemented on the all-species SNP matrix in order to infer recombination and population structure based on the presence or absence of shared genomic haplotype regions, as described elsewhere (48). In brief, the SNP matrix was reduced to a pairwise similarity matrix using Chromopainter, within the *fineStructure* program, employing the linkage model, where the underlying model assumes that individuals within populations would share more regions of their genome with each other and have an amount of admixture similar to that seen with individuals from different populations. Identified populations are merged one at a time, using the most likely population merge at each step, to relate populations to each other through a tree.

In order to determine the amount of between-population and within-population variance, we applied analysis of molecular variance (AMOVA) using the GenAlEx program (59). The AMOVA produces an Fst score and a ΦPT score, which is an analogue of Wright’s Fst. A ΦPT = 0 is considered indicative of no genetic differences among populations, and ΦPT =1 indicates 100% genetic variance. Mega (60) was used to calculate the average SNP distance within each identified population (i.e., the number of base substitutions per site from averaging using all genome pairs within each population).

### Divergence time analysis.

To estimate evolutionary rates for all of the *Coccidioides* species, as well as divergence times for *C. immitis* and *C. posadasii*, we employed a Bayesian molecular clock method as implemented in the BEAST v1.8.0 software package (57). Model selection analyses were carried out in PAUP* version 4.0a142 for the complete *Coccidioides* data set and for each of the two species separately, where the Bayesian information criterion results were used to determine the best-fitting models. While the model selection results were in concordance with the maximum likelihood model selection, BEAST currently has a limited selection of nucleotide substitution models available. Of the models available for use in BEAST, the GTR plus G model was found to be the best-fitting model for both the *Coccidioides* and *C. immitis* datasets, while the HKY plus G model was determined to be the best-fitting model for the *C. posadasii* data set. Because only variable sites were included in this analysis, we corrected for the invariant sites by specifying a Constant Patterns model in the Patterns List of the BEAST xml file. For each data set, the Constant Patterns model includes the number of constant A’s, C’s, T’s, and G’s. Given the high number of SNPs that compose the data set, the use of path sampling (61) and stepping stone (62) sampling marginal-likelihood estimators was computationally prohibitive, so we instead employed a modified version of the Akaike’s information criterion (AICM) to determine the best-fitting clock and demographic model combinations (63, 64). Model comparison analyses indicated that the combination of the strict molecular clock and the constant population models best fit all three datasets.

The strict clock model was used to infer the time scale and mutation rates through the incorporation of two separate internal calibrations on the node defining the speciation event. The first speciation calibration date, from Sharpton et al. (8), was estimated to be 5.1 MYA through the use of a prior fossil-based calibration point. Previously, Fisher et al. (9) had calculated a deeper speciation time of 12.8 MYA by determining microsatellite flanking sequence genetic distances. A normal prior age distribution with a standard deviation (SD) of 0.25 MYA was used for each of the full *Coccidioides* analyses. For the within-species BEAST analyses, the mean TMRCA time points from the 5.1 MYA run for *C. immitis* (normal prior age distribution [mean, 365,700 years ago; SD, 30,000]) and *C. posadasii* (normal prior age distribution [mean, 818,100 years ago; SD, 50,000]) were used as calibration points. Importantly, the normal prior age distribution was applied to all calibration points in this study, as calibrations based on molecular studies or those that are secondary calibrations have added uncertainty, and data corresponding to that uncertainty in age are equally distributed about the mean.

Visual trace inspection and calculation of effective sample sizes were conducted using Tracer (65), confirming Markov chain Monte Carlo (MCMC) mixing within chains and also among chains for the full *Coccidioides* runs. However, in agreement with the low consistency indices calculated during the parsimony analyses, we did not visualize among-chain convergences within species. Consistent with high levels of recombination, the lack of among-chain convergence indicates that multiple phylogenies can explain the evolutionary history within *Coccidioides* species. For each data set, four independent MCMC chains were run for 100 million generations each, with parameters and trees drawn from the posterior every 10,000 steps. LogCombiner (57) was used to merge the samples from each chain. For each of the full *Coccidioides* analyses and for the *C. immitis* analysis, the first 20% of each chain was discarded as a burn in, and then each chain was resampled every 30,000 steps. For the *C. posadasii* analysis, within-chain convergence occurred later in two of the four runs, the first 50% and 80% of the runs were discarded, and all four chains were resampled every 20,000 steps. The posterior mean and 95% confidence intervals have been reported for evolutionary rates and time to most recent common ancestor estimates for select nodes.

### Mating type locus distribution.

The *MAT1-1* gene from *C. immitis* (EF472259.1) and the *MAT1-2* gene from *C. posadasii* (EF472258.1) were used as query sequences for sexual idiomorphic identification in *Coccidioides* (66). Protein sequences from *MAT* genes were searched against each assembled genome analyzed here via tBLASTn tool (67), and retrieved alignments were manually inspected in order to check the completeness of each *Coccidioides* MAT gene identified. The idiomorphic distributions were assigned to each clade deduced by ML tree analyses. Mating type distribution significances were tested for deviation from the expected *MAT* ratio of 1:1 using a chi-square test and Microsoft Excel. Uneven *MAT* distribution results with a *P* value of <0.05 were considered significant.

### Nucleotide sequence accession number.

All WGS data files have been deposited in the NCBI Sequence Read Archive (http://www.ncbi.nlm.nih.gov/bioproject; project number PRJNA274372).

## SUPPLEMENTAL MATERIAL

Figure S1  Phylogenetic analysis of *C. immitis* and *C. posadasii* isolates from all known regions of endemicity. Maximum parsimony phylogenetic analysis was performed on WGS data from 81 *Coccidioides* genomes, including 12 publically available genomes: 22 *C. immitis* genomes and 59 *C. posadasii* genomes. The shading of clades correlates with clades in Fig. 1. The analysis identified 128,871 shared SNPs, with 70,419 being parsimony informative, with a consistency index of 0.358 and a retention index (RI) of 0.847. The tree shown is midpoint rooted. Branch lengths represent numbers of SNPs between taxa, with the unit bar in the figure. Download Figure S1, TIF file, 1.5 MB

Figure S2  Maximum likelihood SNP phylogenetic analysis of *C. immitis* and *C. posadasii* isolates. Maximum likelihood analysis was performed on WGS data from 69 *Coccidioides* genomes using the TIM3 plus ASC plus R10 model with the program IQ-TREE with 1,000-bootstrap support. The shading of clades correlates with clades in Fig. 1. The tree is midpoint rooted, and San Diego_1 assembly was used as the reference. Download Figure S2, TIF file, 1.5 MB

Figure S3  Maximum parsimony phylogenetic analysis of *C. posadasii* with publically available genomes. Maximum parsimony phylogenetic analysis was performed on WGS data from 58 *C. posadasii* genomes, including 7 publically available genomes. The shading of clades correlates with clades in Fig. 1. The analysis identified 142,261 total SNPs, with 67,166 parsimony informative SNPs, a consistency index of 0.228, and a retention index (RI) of 0.36. The tree shown is rooted using the Guatemalan clade based on data shown in Fig. S1. The unit bar in the figure represents SNPs. Download Figure S3, TIF file, 1.5 MB

Figure S4  Maximum likelihood phylogenetic analysis of *C. posadasii* isolates. Maximum likelihood analysis was performed on WGS data from 51 *C. posadasii* genomes. IQ TREE identified the TVM plus ASC plus R9 model as the correct model to use, and 1,000-bootstrap support was performed. The shading of clades correlates with clades shown in Fig. 1. Numbers on branches represent bootstrap values. The tree is midpoint rooted. Download Figure S4, TIF file, 1.5 MB

Figure S5  Phylogenetic network of *C. posadasii*. A neighbor-net representation of the relationships among the 51 *C. posadasii* isolates in Fig. 3 based on SNP data, using the uncorrected P distance transformation, is shown. Each band of parallel edges indicates a split. Splits of major phylogeographically clustered subpopulations are shaded according to the key. Download Figure S5, TIF file, 1.5 MB

Figure S6  Population structure analysis of *C. immitis* isolates. Bayesian analysis of *C. immitis* population structure was carried out using BAPS 6.0, 2 fixed genetically diverged groups previously established by phylogenetic inferences (Fig. S2), and 10 replicates. Admixture graphs of the two identified *C. immitis* mixtures (populations) were plotted using 200 simulations, and the percentage of genetic composition from each isolate was plotted. Download Figure S6, TIF file, 1.5 MB

Figure S7  Maximum likelihood phylogenetic analysis of *C. immitis* isolates. Maximum likelihood analysis was performed on WGS data from 18 *C. immitis* isolates using the TVM plus ASC plus R7 model with 1,000-bootstrap support. Numbers on branches represent bootstrap values. The tree was midpoint rooted. Download Figure S7, TIF file, 1.5 MB

Figure S8  Maximum parsimony phylogenetic analysis of *C. immitis* isolates. Maximum parsimony analysis was performed on WGS data from 18 *C. immitis* isolates. A total of 64,096 SNPs were identified; 31,372 were parsimony informative. The consistency index value was 0.346, and the RI value was 0.384. The tree was rooted using Coahuila 1, based on the tree shown in Fig. S1. Download Figure S8, TIF file, 1.5 MB

Figure S9  Mating type distribution of *Coccidioides* subpopulations. We analyzed the mating type background from each of the available *Coccidioides* genomes sequenced so far. The sequences of the *MAT1-1* gene from *C. immitis* (EF472259.1) and the *MAT1-2* gene from *C. posadasii* (EF472258.1) were used as query sequences for sexual idiomorphic identification in *Coccidioides*. Mating types were counted for each of the *Coccidioides* clades diagnosed by phylogenomic analysis. Mating type distribution significances were tested for deviation from the expected *MAT* ratio of 1:1 using a chi-square test. Uneven *MAT* distribution data with *P* values of < 0.05 were considered significant. Download Figure S9, TIF file, 1.5 MB

Table S1  Analyzed *Coccidioides* isolate list. Table S1, DOCX file, 0.2 MB
