# Supplementary figures and images for: Local Population Structure and Patterns of Western Hemisphere Dispersal for Coccidioides spp., the Fungal Cause of Valley Fever
Source: mBio. 2016 Apr 26;7(2):e00550-16. doi: 10.1128/mBio.00550-16 (PMC4850269; doi:10.1128/mBio.00550-16)

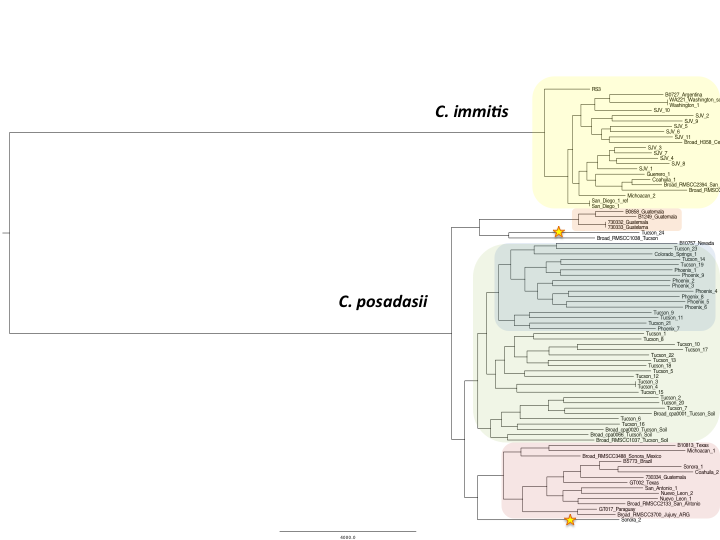

Supplement: Figure S1 — Phylogenetic analysis of C. immitis and C. posadasii isolates from all known regions of endemicity. Maximum parsimony phylogenetic analysis was performed on WGS data from 81 Coccidioides genomes, including 12 publically available genomes: 22 C. immitis genomes and 59 C. posadasii genomes. The shading of clades correlates with clades in Fig. 1. The analysis identified 128,871 shared SNPs, with 70,419 being parsimony informative, with a consistency index of 0.358 and a retention index (RI) of 0.847. The tree shown is midpoint rooted. Branch lengths represent numbers of SNPs between taxa, with the unit bar in the figure. Download [file mbo002162796sf1.tif]

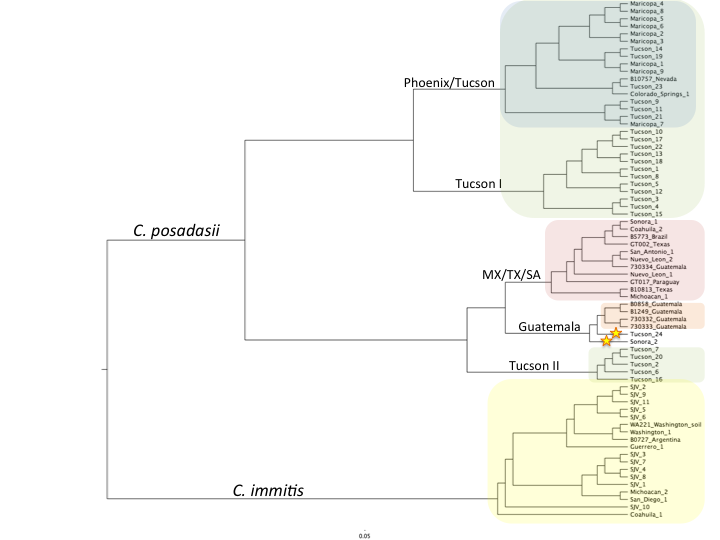

Supplement: Figure S2 — Maximum likelihood SNP phylogenetic analysis of C. immitis and C. posadasii isolates. Maximum likelihood analysis was performed on WGS data from 69 Coccidioides genomes using the TIM3 plus ASC plus R10 model with the program IQ-TREE with 1,000-bootstrap support. The shading of clades correlates with clades in Fig. 1. The tree is midpoint rooted, and San Diego_1 assembly was used as the reference. Download [file mbo002162796sf2.tif]

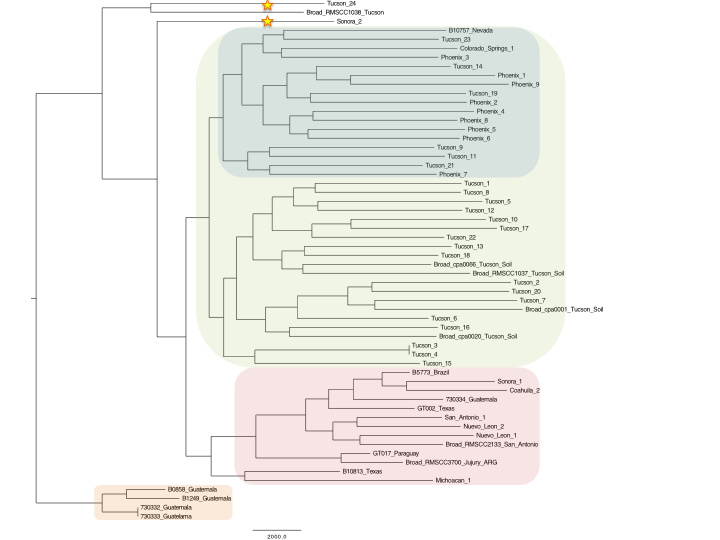

Supplement: Figure S3 — Maximum parsimony phylogenetic analysis of C. posadasii with publically available genomes. Maximum parsimony phylogenetic analysis was performed on WGS data from 58 C. posadasii genomes, including 7 publically available genomes. The shading of clades correlates with clades in Fig. 1. The analysis identified 142,261 total SNPs, with 67,166 parsimony informative SNPs, a consistency index of 0.228, and a retention index (RI) of 0.36. The tree shown is rooted using the Guatemalan clade based on data shown in Fig. S1. The unit bar in the figure represents SNPs. Download [file mbo002162796sf3.tif]

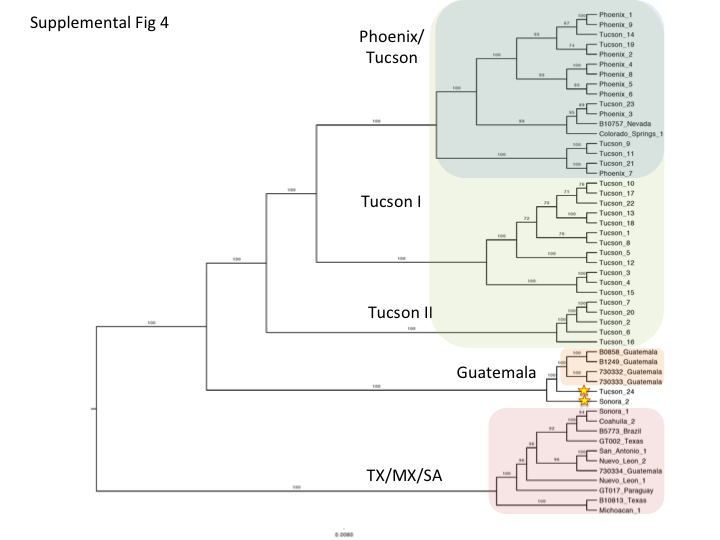

Supplement: Figure S4 — Maximum likelihood phylogenetic analysis of C. posadasii isolates. Maximum likelihood analysis was performed on WGS data from 51 C. posadasii genomes. IQ TREE identified the TVM plus ASC plus R9 model as the correct model to use, and 1,000-bootstrap support was performed. The shading of clades correlates with clades shown in Fig. 1. Numbers on branches represent bootstrap values. The tree is midpoint rooted. Download [file mbo002162796sf4.tif]

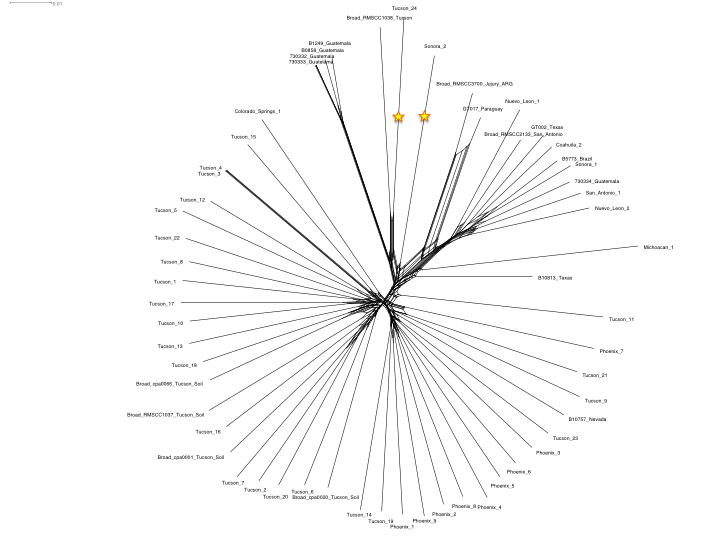

Supplement: Figure S5 — Phylogenetic network of C. posadasii. A neighbor-net representation of the relationships among the 51 C. posadasii isolates in Fig. 3 based on SNP data, using the uncorrected P distance transformation, is shown. Each band of parallel edges indicates a split. Splits of major phylogeographically clustered subpopulations are shaded according to the key. Download [file mbo002162796sf5.tif]

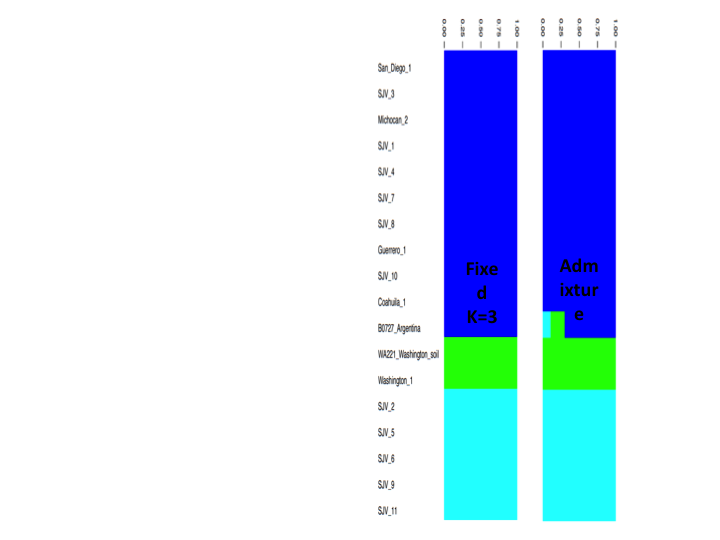

Supplement: Figure S6 — Population structure analysis of C. immitis isolates. Bayesian analysis of C. immitis population structure was carried out using BAPS 6.0, 2 fixed genetically diverged groups previously established by phylogenetic inferences (Fig. S2), and 10 replicates. Admixture graphs of the two identified C. immitis mixtures (populations) were plotted using 200 simulations, and the percentage of genetic composition from each isolate was plotted. Download [file mbo002162796sf6.tif]

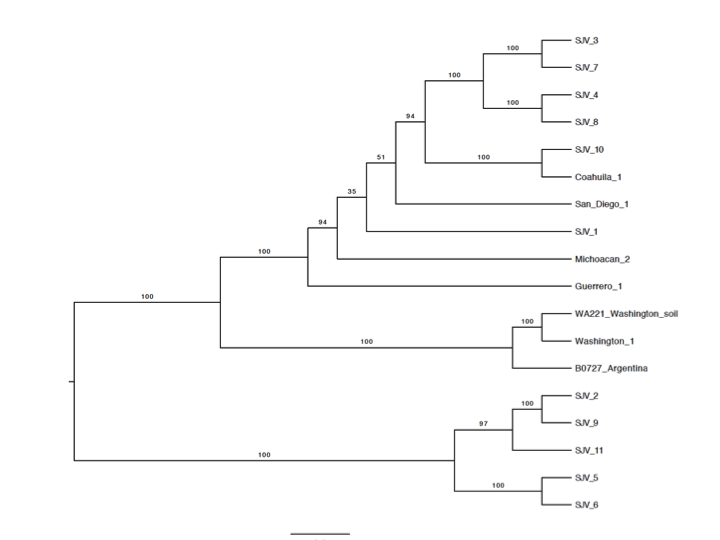

Supplement: Figure S7 — Maximum likelihood phylogenetic analysis of C. immitis isolates. Maximum likelihood analysis was performed on WGS data from 18 C. immitis isolates using the TVM plus ASC plus R7 model with 1,000-bootstrap support. Numbers on branches represent bootstrap values. The tree was midpoint rooted. Download [file mbo002162796sf7.tif]

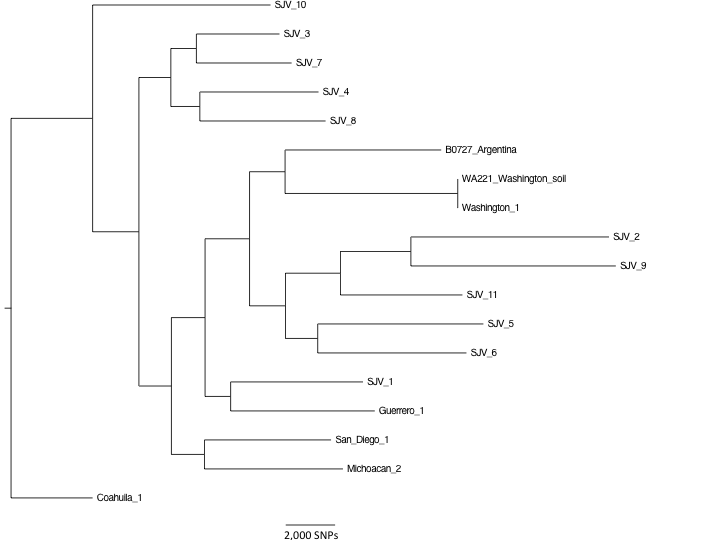

Supplement: Figure S8 — Maximum parsimony phylogenetic analysis of C. immitis isolates. Maximum parsimony analysis was performed on WGS data from 18 C. immitis isolates. A total of 64,096 SNPs were identified; 31,372 were parsimony informative. The consistency index value was 0.346, and the RI value was 0.384. The tree was rooted using Coahuila 1, based on the tree shown in Fig. S1. Download [file mbo002162796sf8.tif]

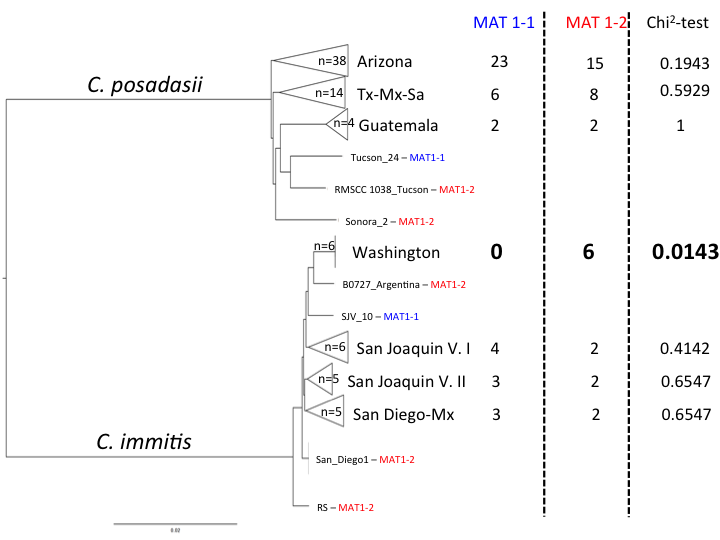

Supplement: Figure S9 — Mating type distribution of Coccidioides subpopulations. We analyzed the mating type background from each of the available Coccidioides genomes sequenced so far. The sequences of the MAT1-1 gene from C. immitis (EF472259.1) and the MAT1-2 gene from C. posadasii (EF472258.1) were used as query sequences for sexual idiomorphic identification in Coccidioides. Mating types were counted for each of the Coccidioides clades diagnosed by phylogenomic analysis. Mating type distribution significances were tested for deviation from the expected MAT ratio of 1:1 using a chi-square test. Uneven MAT distribution data with P values of < 0.05 were considered significant. Download [file mbo002162796sf9.tif]
