# Supplementary material for: Local Population Structure and Patterns of Western Hemisphere Dispersal for Coccidioides spp., the Fungal Cause of Valley Fever
Source: mBio. 2016 Apr 26;7(2):e00550-16. doi: 10.1128/mBio.00550-16 (PMC4850269; doi:10.1128/mBio.00550-16)
Supplement: Table S1 — Analyzed Coccidioides isolate list. [file mbo002162796st1.docx]

Supplemental Table 1. Analyzed *Coccidioides* isolate list

| **Tree ID** | **Species ID** | **Locale**  **City/County/State** | **Geographic**  **Location** | **Year** | **Figure**  **Appearance** | **Average**  **X Coverage** | **Percent mapped to reference at 10x coverage** |
| --- | --- | --- | --- | --- | --- | --- | --- |
| Tucson_1 | *C. posadasii* | Tucson/Pima | Arizona | 1990 | 1,2,4,5,S1-S5, S9 | 62 | 86.51% |
| Tucson_2 | *C. posadasii* | Tucson/Pima | Arizona | ~ | 1,2,4,5,S1-S5, S9 | 66 | 86.83% |
| Tucson_3 | *C. posadasii* | Tucson/Pima | Arizona | 1991 | 1,2,4,5,S1-S5, S9 | 236 | 89.44% |
| Tucson_4 | *C. posadasii* | Tucson/Pima | Arizona | 1991 | 1,2,4,5,S1-S5, S9 | 100 | 87.66% |
| Tucson_5 | *C. posadasii* | Tucson/Pima | Arizona | 1991 | 1,2,4,5,S1-S5, S9 | 88 | 87.04% |
| Tucson_6 | *C. posadasii* | Tucson/Pima | Arizona | 1991 | 1,2,4,5,S1-S5, S9 | 53 | 85.89% |
| Tucson_7 | *C. posadasii* | Tucson/Pima | Arizona | 1991 | 1,2,4,5,S1-S5, S9 | 106 | 88.19% |
| Tucson_8 | *C. posadasii* | Tucson/Pima | Arizona | ~ | 1,2,4,5,S1-S5, S9 | 101 | 87.60% |
| Tucson_9 | *C. posadasii* | Tucson/Pima | Arizona | 1988 | 1,2,4,5,S1-S5, S9 | 115 | 87.68% |
| Tucson_10 | *C. posadasii* | Tucson/Pima | Arizona | 1988 | 1,2,4,5,S1-S5, S9 | 100 | 87.42% |
| Tucson_11 | *C. posadasii* | Tucson/Pima | Arizona | 1988 | 1,2,4,5,S1-S5, S9 | 133 | 88.40% |
| Tucson_12 | *C. posadasii* | Tucson/Pima | Arizona | 1988 | 1,2,4,5,S1-S5, S9 | 105 | 87.61% |
| Tucson_13 | *C. posadasii* | Tucson/Pima | Arizona | 1988 | 1,2,4,5,S1-S5, S9 | 53 | 87.21% |
| Tucson_14 | *C. posadasii* | Tucson/Pima | Arizona | 1989 | 1,2,4,5,S1-S5, S9 | 36 | 84.96% |
| Tucson_15 | *C. posadasii* | Tucson/Pima | Arizona | 1989 | 1,2,4,5,S1-S5, S9 | 34 | 85.05% |
| Tucson_16 | *C. posadasii* | Tucson/Pima | Arizona | 1989 | 1,2,4,5,S1-S5, S9 | 69 | 86.83% |
| Tucson_17 | *C. posadasii* | Tucson/Pima | Arizona | 1986 | 1,2,4,5,S1-S5, S9 | 56 | 85.77% |
| Tucson_18 | *C. posadasii* | Tucson/Pima | Arizona | 1987 | 1,2,4,5,S1-S5, S9 | 37 | 85.15% |
| Tucson_19 | *C. posadasii* | Tucson/Pima | Arizona | 1987 | 1,2,4,5,S1-S5, S9 | 71 | 86.86% |
| Tucson_20 | *C. posadasii* | Tucson/Pima | Arizona | 1987 | 1,2,4,5,S1-S5, S9 | 53 | 86.13% |
| Tucson_21 | *C. posadasii* | Tucson/Pima | Arizona | 1988 | 1,2,4,5,S1-S5, S9 | 131 | 88.17% |
| Tucson_22 | *C. posadasii* | Tucson/Pima | Arizona | 1988 | 1,2,4,5,S1-S5, S9 | 33 | 84.72% |
| Tucson_23 | *C. posadasii* | Tucson/Pima | Arizona | 1976 | 1,2,4,5,S1-S5, S9 | 59 | 86.02% |
| San_Antonio_1 | *C. posadasii* | San Antonio | Texas | ~ | 1,2,4,5,S1-S5, S9 | 81 | 87.04% |
| Colorado_Springs_1 | *C. posadasii* | Colorado Springs | Colorado | 1997 | 1,2,4,5,S1-S5, S9 | 68 | 86.38% |
| Nuevo_Leon_1 | *C. posadasii* | Nuevo Leon | Cent. Mexico | ~ | 1,2,4,5,S1-S5, S9 | 74 | 85.85% |
| Nuevo_Leon_2 | *C. posadasii* | Nuevo Leon | Cent. Mexico | ~ | 1,2,4,5,S1-S5, S9 | 95 | 86.30% |
| Michoacan_1 | *C. posadasii* | Michoacán | South Mexico | ~ | 1,2,4,5,S1-S5, S9 | 92 | 86.55% |
| Sonora_1 | *C. posadasii* | Sonora | North Mexico | ~ | 1,2,4,5,S1-S5, S9 | 46 | 85.35% |
| Sonora_2 | *C. posadasii* | Sonora | North Mexico | ~ | 1,2,4,5,S1-S5, S9 | 33 | 84.84% |
| Tucson_24 | *C. posadasii* | Tucson/Pima | Arizona | 1991 | 1,2,4,5,S1-S5, S9 | 167 | 88.53% |
| Coahuila_2 | *C. posadasii* | Coahuila | North Mexico | ~ | 1,2,4,5,S1-S5, S9 | 82 | 87.27% |
| Phoenix_1 | *C. posadasii* | Phoenix/Maricopa | Arizona | 2002 | 1,2,4,5,S1-S5, S9 | 39 | 85.12% |
| Phoenix _2 | *C. posadasii* | Phoenix/Maricopa | Arizona | 2003 | 1,2,4,5,S1-S5, S9 | 84 | 86.82% |
| Phoenix _3 | *C. posadasii* | Phoenix/Maricopa | Arizona | 2002 | 1,2,4,5,S1-S5, S9 | 74 | 86.30% |
| Phoenix _4 | *C. posadasii* | Phoenix/Maricopa | Arizona | 2003 | 1,2,4,5,S1-S5, S9 | 47 | 85.12% |
| Phoenix _5 | *C. posadasii* | Phoenix/Maricopa | Arizona | 2002 | 1,2,4,5,S1-S5, S9 | 66 | 86.65% |
| Phoenix _6 | *C. posadasii* | Phoenix/Maricopa | Arizona | 2002 | 1,2,4,5,S1-S5, S9 | 37 | 85.22% |
| Phoenix _7 | *C. posadasii* | Phoenix/Maricopa | Arizona | 2002 | 1,2,4,5,S1-S5, S9 | 50 | 85.46% |
| Phoenix _8 | *C. posadasii* | Phoenix/Maricopa | Arizona | 2002 | 1,2,4,5,S1-S5, S9 | 40 | 84.95% |
| Phoenix _9 | *C. posadasii* | Phoenix/Maricopa | Arizona | 2002 | 1,2,4,5,S1-S5, S9 | 49 | 85.66% |
| GT017_Paraguay | *C. posadasii* | Paraguay | Paraguay | ~ | 1,2,4,5,S1-S5, S9 | 39 | 83.51% |
| GT002_Texas | *C. posadasii* | Midland | Texas | 1947 | 1,2,4,5,S1-S5, S9 | 73 | 85.13% |
| B0858_Guatemala | *C. posadasii* | Guatemala | Guatemala | 1967 | 1,2,4,5,S1-S5, S9 | 64 | 85.53% |
| 730332_Guatemala | *C. posadasii* | Guatemala | Guatemala | 2014 | 1,2,4,5,S1-S5, S9 | 34 | 83.86% |
| 730333_Guatemala | *C. posadasii* | Guatemala | Guatemala | 2014 | 1,2,4,5,S1-S5, S9 | 31 | 83.05% |
| 730334_Guatemala | *C. posadasii* | Guatemala | Guatemala | 2014 | 1,2,4,5,S1-S5, S9 | 44 | 83.88% |
| B10757_Nevada | *C. posadasii* | Nevada | Nevada | 2014 | 1,2,4,5,S1-S5, S9 | 50 | 84.68% |
| B10813_Tx | *C. posadasii* | Texas | Texas | 2014 | 1,2,4,5,S1-S5, S9 | 88 | 85.20% |
| B1249_Guatemala | *C. posadasii* | Guatemala | Guatemala | 1971 | 1,2,4,5,S1-S5, S9 | 62 | 85.36% |
| B5773_Brazil | *C. posadasii* | Brazil | Brazil | 1997 | 1,2,4,5,S1-S5, S9 | 53 | 84.04% |
| RMSCC 2133 | *C. posadasii* | San Antonio | Texas | ~ | 4,S1,S3,S5 | ~ | ~ |
| RMSCC 3700 | *C. posadasii* | Jujuy | Argentina | ~ | 4,S1,S3,S5 | ~ | ~ |
| RMSCC 3488 | *C. posadasii* | Sonora | Mexico | ~ | 4,S1,S3,S5 | ~ | ~ |
| RMSCC 1038 | *C. posadasii* | Tucson | Arizona | ~ | 4,S1,S3,S5 | ~ | ~ |
| RMSCC 1040 | *C. posadasii* | Arizona | Arizona | ~ | 4,S1,S3,S5 | ~ | ~ |
| RMSCC 1037_Soil | *C. posadasii* | Tucson | Arizona | ~ | 4,S1,S3,S5 | ~ | ~ |
| CPA 0001_Soil | *C. posadasii* | Tucson | Arizona | ~ | 4,S1,S3,S5 | ~ | ~ |
| CPA 0020_Soil | *C. posadasii* | Tucson | Arizona | ~ | 4,S1,S3,S5 | ~ | ~ |
| CPA 0066_Soil | *C. posadasii* | Tucson | Arizona | ~ | 4,S1,S3,S5 | ~ | ~ |
| B0727_Argentina | *C. immitis* | Buenos Aries | Argentina | 1959 | 1,3-5,S1,S2,S6-9 | 42 | 93.69% |
| Michoacan_2 | *C. immitis* | Michoacan | South Mexico | ~ | 1,3-5,S1,S2,S6-9 | 22 | 87.61% |
| Guerrero_1 | *C. immitis* | Guerrero | South Mexico | ~ | 1,3-5,S1,S2,S6-9 | 33 | 93.49% |
| San_Diego_1 | *C. immitis* | San Diego | California | ~ | 1,3-5,S1,S2,S6-9 | 52 | 98.30% |
| Coahuila_1 | *C. immitis* | Coahuila | Mexico | ~ | 1,3-5,S1,S2,S6-9 | 82 | 95.50% |
| SJV_1 | *C. immitis* | San Joaquin Valley | California | ~ | 1,3-5,S1,S2,S6-9 | 24 | 92.58% |
| SJV_2 | *C. immitis* | San Joaquin Valley | California | ~ | 1,3-5,S1,S2,S6-9 | 69 | 93.78% |
| SJV_3 | *C. immitis* | San Joaquin Valley | California | ~ | 1,3-5,S1,S2,S6-9 | 22 | 85.65% |
| SJV_4 | *C. immitis* | San Joaquin Valley | California | ~ | 1,3-5,S1,S2,S6-9 | 90 | 95.18% |
| SJV_5 | *C. immitis* | San Joaquin Valley | California | ~ | 1,3-5,S1,S2,S6-9 | 57 | 94.01% |
| SJV_6 | *C. immitis* | San Joaquin Valley | California | ~ | 1,3-5,S1,S2,S6-9 | 19 | 89.33% |
| SJV_7 | *C. immitis* | San Joaquin Valley | California | ~ | 1,3-5,S1,S2,S6-9 | 100 | 94.64% |
| SJV_8 | *C. immitis* | San Joaquin Valley | California | ~ | 1,3-5,S1,S2,S6-9 | 33 | 93.77% |
| SJV_9 | *C. immitis* | San Joaquin Valley | California | ~ | 1,3-5,S1,S2,S6-9 | 26 | 92.87% |
| SJV_10 | *C. immitis* | San Joaquin Valley | California | ~ | 1,3-5,S1,S2,S6-9 | 73 | 95.28% |
| SJV_11 | *C. immitis* | San Joaquin Valley | California | ~ | 1,3-5,S1,S2,S6-9 | 37 | 92.86% |
| Washington_1 | *C. immitis* | SE Washington | Washington | 2010 | 1,3-5,S1,S2,S6-9 | 75 | 93.90% |
| WA221-Soil | *C. immitis* | SE Washington | Washington | 2014 | 1,3-5,S1,S2,S6-9 | 30 | 92.12% |
| CDC202_soil | *C. immitis* | SE Washington | Washington | 2010 | S9 | 63 | ~ |
| CDC205_soil | *C. immitis* | SE Washington | Washington | 2010 | S9 | 63 | ~ |
| CDC211_soil | *C. immitis* | SE Washington | Washington | 2010 | S9 | 60 | ~ |
| CDC212_soil | *C. immitis* | SE Washington | Washington | 2010 | S9 | 67 | ~ |
| RS | *C. immitis* | Central CA | California | ~ | 4,S1,S9 | ~ | ~ |
| H538.4 | *C. immitis* | Central CA | California | ~ | 4,S1 | ~ | ~ |
| RMSCC 2394 | *C. immitis* | San Diego | California | ~ | 4,S1 | ~ | ~ |
| RMSCC 3703 | *C. immitis* | San Diego | California | ~ | 4,S1 | ~ | ~ |
